# Multiple Bony Injuries on Bone Scan in a Case of Unsuspected Child Abuse

**DOI:** 10.1155/2017/3015941

**Published:** 2017-06-27

**Authors:** Ya-Wen Chuang, Chien-Chin Hsu, Chin-Chuan Chang, Chia-Yang Lin, Hsiu-Lan Chu, Ying-Fong Huang, Yu-Chang Tyan

**Affiliations:** ^1^Department of Nuclear Medicine, Kaohsiung Medical University Hospital, Kaohsiung, Taiwan; ^2^Department of Nuclear Medicine, Kaohsiung Chang Gung Memorial Hospital, Chang Gung University College of Medicine, Taoyuan City, Taiwan; ^3^Department of Medical Imaging and Radiological Sciences, College of Health Science, Kaohsiung Medical University, Kaohsiung, Taiwan; ^4^Center for Infectious Disease and Cancer Research, Kaohsiung Medical University, Kaohsiung, Taiwan; ^5^Institute of Medical Science and Technology, National Sun Yat-sen University, Kaohsiung, Taiwan; ^6^Graduate Institute of Medicine, College of Medicine, Kaohsiung Medical University, Kaohsiung, Taiwan; ^7^Department of Medical Research, Kaohsiung Medical University Hospital, Kaohsiung, Taiwan

## Abstract

This case is described of an eleven-month-old infant with lower limbs swelling and the left elbow skeletal malformation following a fall. The radionuclide bone scan was performed to exclude bone infection or congenital skeletal anomaly. The images unexpectedly showed multiple increased radioactive foci throughout the whole body. It was a strong probability of child abuse. All lesions are readily apparent on the following plain film radiographs and MRI.

## 1. Introduction

The battered child syndrome consists of a constellation of signs that may be either apparent or covert. Many patterns of injury have been described in the child abuse syndrome. Bone scintigraphy is a valuable imaging modality in the examination of the battered child. It is often used to evaluate skeletal trauma and identify fractures which previously would have been ignored [1–5].

## 2. Case Report

**Figure 1:**
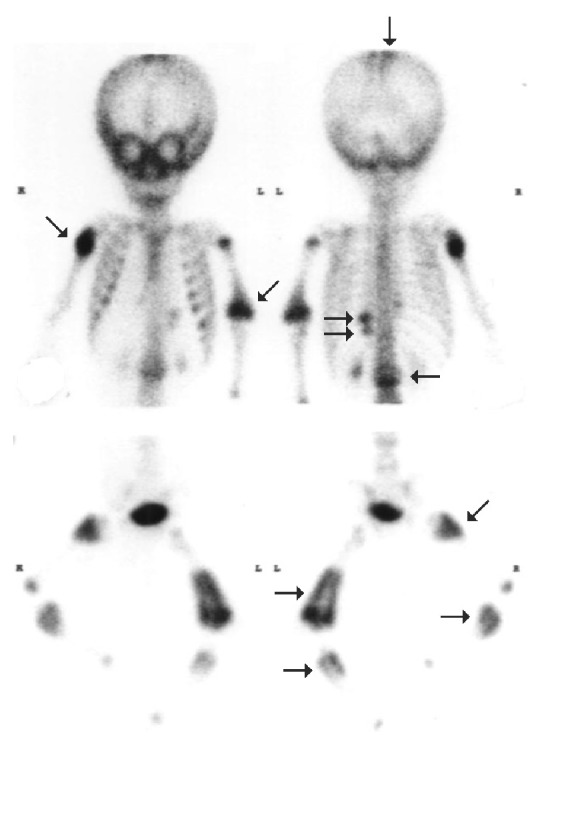
Bone scan: multiple increased radioactive foci throughout the whole body (arrows) were accidentally found. There was a strong probability of child abuse.

**Figure 2:**
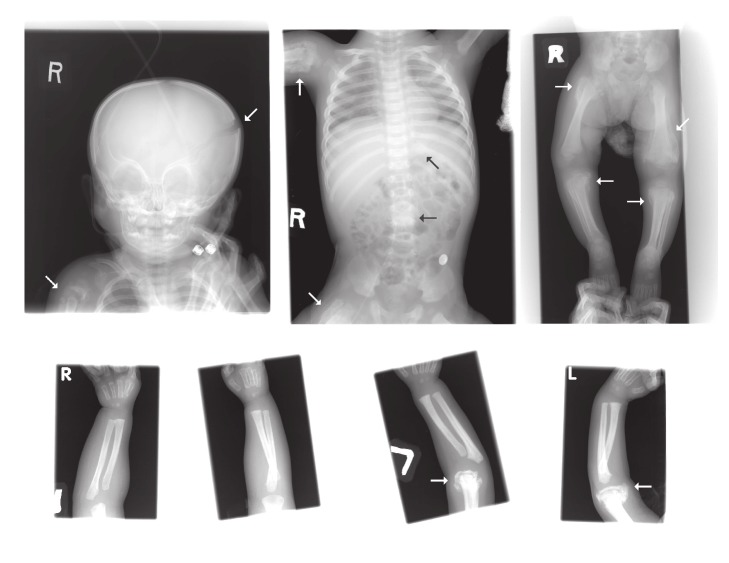
X-ray: a series of plain film radiographs demonstrated calvarial fracture lines at left temporoparietal region, bony anomaly of the spine, multiple old fractures with callus formation involving posterior aspect of left 10, 11th ribs, right proximal humerus, bilateral proximal femurs, and metaphyses of tibias (arrows). Adjacent soft tissue swelling is evident. The above findings were consistent with child abuse.

An eleven-month-old infant weighted 1780 g with a premature birth age of 34 weeks was in the intensive care for seizure attack, aspiration pneumonia, and subdural and subarachnoid hemorrhage. He developed progressive lower limbs swelling and his left elbow skeletal malformation following a fall. To protect the privacy of our patients, their full transcripts are not openly available. The radionuclide bone scan with 185 MBq (5 mCi) Tc-99m MDP was performed for an evaluation of suspicious malunion fracture, bone infection, or preexisting medical conditions because metabolic disorders and bone diseases may make a child's bones more vulnerable to fracture [6]. Multiple increased radioactive foci throughout the whole body (Figure 1) were unexpectedly found. There was a strong probability of child abuse. A series of plain film radiographs demonstrated calvarial fracture lines at left temporoparietal region, bony anomaly of the spine, multiple old fractures with callus formation involving posterior aspect of left 10th, 11th ribs, right proximal humerus, bilateral proximal femurs, and metaphyses of tibias (Figure 2). The above findings were also consistent with child abuse. Radiographic skeletal survey and radionuclide images are complementary procedures for diagnosis and documenting this type of injury [7].

**Figure 3:**
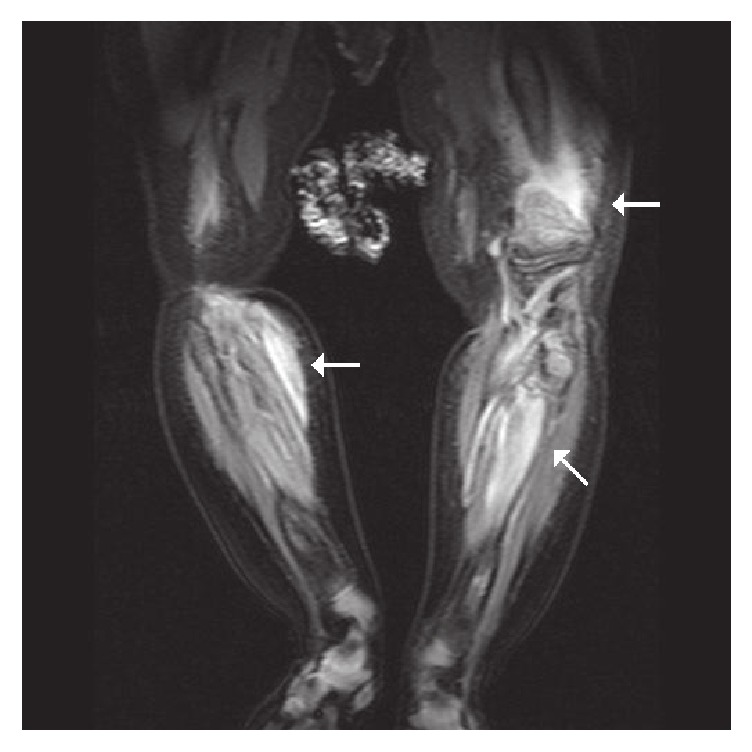
MRI of both lower legs: the T2-weighted coronal images show metaphyseal fractures involving distal left femur and proximal bilateral tibias with extensive periosteal hemorrhage or edema, favored child abuse.

**Figure 4:**
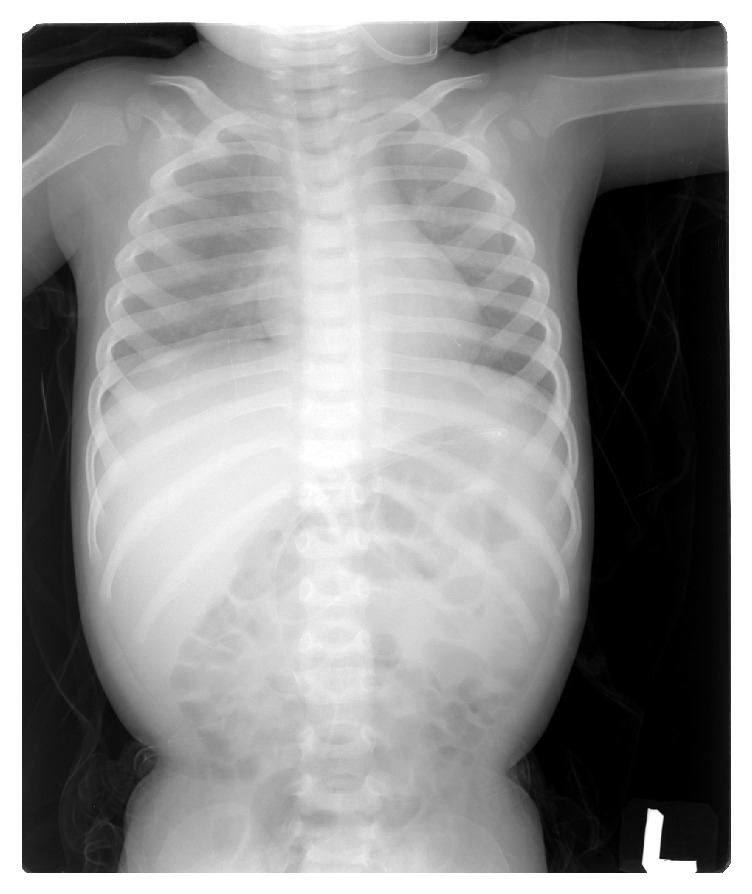
X-ray: the previous multiple bony fractures with callus formation are not prominent on the following plain film radiograph 6 months later.

Adjacent soft tissue swelling was evident. The above findings were also consistent with child abuse. Radiographic skeletal survey and radionuclide images are complementary procedures for diagnosis and documenting this type of injury (Figure 3). The magnetic resonance imaging (MRI) was performed for diagnosing whenever typical skeletal injuries associated with extraskeletal injuries [8]. The MRI T2-weighted coronal images showing metaphyseal fractures of distal left femur and proximal bilateral tibias with extensive periosteal hemorrhage or edema favored child abuse, too. This case was reported and social workers dealt with this event. The previous multiple bony fractures with callus formation were not prominent on the following plain film radiograph, which was taken 6 months later (Figure 4).

## 3. Discussion

The estimated incidence of reported child abuse has increased from 3% in 1985 to 4.5% in 1992 [7]. The incidence of skeletal injury in these children is approximately 20% and is more common among those under 1 year of age. Children older than 3 years of age tend to have predominantly soft tissue injury. Cerebral injury is common at any age. The fractures are usually multiple, involving the long bones, skull, vertebrae, ribs, and facial bones in addition to frequently showing different stages of healing.

Bone scintigraphy is a valuable imaging modality in the examination of these young children, especially in detecting injury in ribs, costovertebral junctions, hands, feet, spine, and diaphyses of long bones [2, 9]. Child abuse should be considered when diagnosing increased lone bone uptake on bone scintigraphy, which may indicate nonaccidental trauma.

The combination of bone scan and X-ray with experienced hands can reduce the false-negative rate from 12.3% to 0.8%. Although the bone scan may be positive as early as 7 hours after injury, the child is usually brought to a hospital so late that the bone healing has begun [10].

The image modalities play a key role in the investigation and documentation of the battered child syndrome. The primary diagnostic imaging study in suspected child abuse is either a bone scan and X-ray series or a complete radiographic skeletal survey by X-ray series in babies and infants [11]. Skeletal survey and bone scintigraphy are complementary studies in the evaluation of nonaccidental injury and should both be performed in cases of suspected child abuse [7, 12, 13]. Further studies should be undertaken in this circumstance to search for coexisting injuries, especially as the history and mechanism of injury may often be unclear. Bone scan may require sedation, and this modality is now less commonly used, especially in the emergent setting [14]. However, in cases where children are potentially being lost to follow-up, this will aid the diagnosis of the majority of fractures during the initial assessment and, therefore, help ensure the safety of the child [3].
